# Exercise-Induced Exerkines Modulate Autophagy: Implications for Interorgan Crosstalk in the Hallmarks of Ageing

**DOI:** 10.3390/ijms27062746

**Published:** 2026-03-18

**Authors:** Qi Deng, Jielun Huang, Cenyi Wang, Jiling Liang

**Affiliations:** 1 Department of Physical Education, Central South University, Changsha 410083, China; 230912010@csu.edu.cn; 2 College of Sports Medicine, Wuhan Sports University, Wuhan 430079, China; 3 School of Physical Education and Sports Science, Soochow University, Suzhou 215006, China

**Keywords:** exerkines, exercise, autophagy, sarcopenia, miRNAs

## Abstract

Population aging and widespread sedentary lifestyles have increased the prevalence of chronic non-communicable diseases, many of which are linked to progressive disruptions of cellular homeostasis. Autophagy, a conserved cellular degradation and recycling pathway, plays a central role in maintaining metabolic flexibility, proteostasis, and organ function. However, aging and physical inactivity impair autophagic regulation, thereby contributing to the development of sarcopenia, cardiovascular diseases, metabolic disorders, and neurodegenerative diseases. Physical exercise is a non-pharmacological intervention that can restore autophagic activity and confer systemic health benefits in multiple preclinical and clinical contexts. Increasing evidence indicates that these benefits are mediated not only by local tissue adaptations but also by complex inter-organ communication. Central to this process are exercise-induced bioactive factors, collectively termed exerkines, including myokines, cardiokines, adipokines, hepatokines, osteokines, and circulating miRNAs. Rather than acting independently, exerkines form an integrated signaling network that fine-tunes autophagic flux across multiple tissues. Exerkine-mediated regulation of autophagy involves key pathways such as AMPK/mTOR, FoxO, SIRT1, ULK1, and TFEB, thereby coordinating energy metabolism, mitochondrial quality control, inflammation, and protein turnover in skeletal muscle, heart, liver, adipose tissue, bone, and the central nervous system. This review summarizes current evidence on representative exerkines and their roles in autophagy-dependent inter-organ crosstalk, highlighting the exercise–exerkine–autophagy axis as a promising target for preventing and managing chronic diseases.

## 1. Introduction

The rapid advancement of economic and social factors has led to increased sedentary behavior, which is associated with an obvious increase in chronic diseases. Globally, one-third of the adult population is inactive, and this trend begins early in life. Sedentary behavior accounts for approximately 9% of premature deaths, primarily due to non-communicable diseases [1]. Physical inactivity is a major risk factor for the development of chronic diseases. As a result, physical activity is recognized as a rational intervention for mitigating various metabolic disorders that affect health [2].

In the past decade, numerous studies have established that skeletal muscle functions as a pivotal protein reserve for the body, playing a critical role in health and survival, regulating metabolism, and contributing to overall quality of life. Initially, the term “myokine” was coined to encompass cytokines and other peptides secreted and released by skeletal muscle. Subsequently, skeletal muscle has also been classified as a secretory organ that releases myokines in response to muscle contraction [3]. Similarly to skeletal muscle, the heart, liver, adipose, and bone tissues secrete various factors, such as cardiokines, hepatokines, adipokines, and osteokines, in response to exercise. These factors, collectively known as “exerkines”, have been shown to facilitate multisystemic benefits [4]. In addition to cytokines and proteins, exercise-induced metabolites, microRNAs (miRNAs), and other RNA species may exert systemic metabolic effects. As a result, exerkines serve as a protective factor against diseases, aging, and the detrimental effects of physical inactivity.

The majority of exerkines are secreted by skeletal muscle, thus playing a crucial role in the exchange of information between metabolic organs, the regulation of metabolism and physiological homeostasis, and influencing the onset and progression of bodily conditions [5,6]. The discovery of exerkines offers a novel approach to elucidating the underlying mechanisms of exercise in the prevention and treatment of chronic diseases. Currently, an increasing number of biotechnology firms and medical institutions are directing their research and development efforts towards the creation of new compounds that can harness the benefits of exercise, augment exercise performance, or simulate exercise. These endeavors are particularly crucial for individuals leading sedentary lifestyles, suffering from chronic diseases, or lacking the motivation to engage in physical activity.

Chronic physical inactivity results in the deficiency and dysfunction of autophagy, exacerbating metabolic, neurodegenerative, cardiovascular, aging, and other age-related diseases [7,8]. In contrast, exercise stimulates autophagy, leading to the recycling of cellular components as part of the adaptive response to exercise. Recent studies have demonstrated that exercise can induce autophagy in various tissues and organs, including skeletal muscle, heart, brain, adipose tissue, pancreas, liver, and bone, indicating that exercise-induced autophagy can occur at a systemic level [9]. Unlike conventional drug therapy, exercise exerts systemic effects by simultaneously targeting multiple organs while also inducing tissue-specific adaptations. Furthermore, autophagy has been shown to ameliorate intracellular environmental disorders, mitigating inflammation and reducing suboptimal protein folding [10]. A comprehensive understanding of exerkines and the autophagic state associated with various health conditions can contribute to the development of novel preventive and therapeutic approaches to enhance physical fitness. This article examines several exerkines involved in autophagy and inter-tissue communication with skeletal muscle in the context of chronic diseases. Specifically, we examine the alterations of these exerkines in response to physical activity. Understanding the intricate interplay between various tissues and skeletal muscle may pave the way for more effective therapeutic interventions aimed at prolonging health span and mitigating chronic ailments.

### Search Strategy and Methodology

To ensure the transparency and quality of this narrative review, we followed the Standards for Reporting Qualitative Research (SRQR) guidelines [11]. A comprehensive literature search was conducted across PubMed, Web of Science, and Embase databases. The search strategy employed combinations of keywords including “exerkines”, “exercise”, “autophagy”, “inter-organ crosstalk”, and “chronic diseases”. Studies were primarily selected based on their relevance to exercise-induced bioactive factors and their mechanistic roles in autophagic regulation across multiple organ systems, with a focus on recent high-quality randomized controlled trials and mechanistic studies. We included original experimental studies (human, animal, or cell models) and high-quality reviews that examined (i) exercise-responsive exerkines and (ii) mechanistic links to autophagy or autophagic flux in at least one tissue. We preferentially considered studies published in English over the last ~15 years, while seminal earlier work was retained when it provided foundational concepts. Studies focusing solely on static autophagy markers without discussion of flux were interpreted with caution and were noted as a limitation where relevant.

## 2. Exercise-Induced Autophagy Confers Health Benefits

It is critical to distinguish between the steady-state level of autophagosomes and autophagic flux, which refers to the dynamic rate of the entire process from formation to lysosomal degradation [12]. Contradictory findings in the current literature, such as those regarding IGF-1, often stem from static measurements of LC3-II or p62 [13]. An increase in LC3-II may reflect either enhanced autophagy induction or a blockade in downstream lysosomal fusion [14,15]. Therefore, dynamic flux assays (e.g., using lysosomal inhibitors) are essential for determining whether an exerkine truly promotes or impairs cellular clearance.

Exercise-induced autophagy was first discovered in the skeletal muscle of mice in 1984 [16], which was subsequently corroborated by the finding that endurance exercise can lead to the degradation of proteins in the liver [17]. In 2011, the activation of autophagy was confirmed in skeletal muscle during hyper-endurance exercise through the increased conversion of LC3-I to LC3-II [18]. Autophagy serves as a primary mechanism for providing an alternative energy source in skeletal muscle during exercise, which is particularly significant for meeting the high demand for oxygen and glucose in tissues. In a low-oxygen and -glucose environment, autophagy plays a crucial role in mitigating excessive fatigue and damage to skeletal muscle, thereby facilitating tissue adaptation to exercise.

Recent studies have extensively reported the initiation of exercise-induced autophagy and cell recycling [19,20]. The process of autophagy in skeletal muscle is triggered by the activation of AMPK and SIRT1, which are responsive to changes in AMP and NAD^+^. This activation leads to the stimulation of the forkhead box O (FoxO) family of transcription factors, resulting in the up-regulation of *Atg* genes by inhibiting the mammalian target of rapamycin complex 1 (mTORC1) and increasing peroxisome-proliferator-activated receptor gamma coactivator-1 alpha (PGC-1α) activity. Additionally, AMPK initiates autophagosome formation through ULK1. During subsequent physical activity, mTORC1 dissociates from the lysosome, resulting in the dephosphorylation of transcription factor EB (TFEB) and subsequent translocation to the nucleus to trigger the transcription of *Atg* genes.

Over the past few years, significant endeavors have been undertaken to investigate exercise-induced autophagy in skeletal muscle, and emerging evidence suggests that exercise may also induce autophagy in other tissues, thereby activating a systemic autophagic response [20]. Exercise can effectively activate autophagy in the liver, heart, adipose tissue, and pancreatic beta cells of wild-type mice, but not in autophagy-deficient mutant mice [21]. Following a 10-week exercise intervention for high-fat diet (HFD)-induced mice, a correlation is observed between the augmentation of *Atg* proteins in hepatocytes and the decrease in lipid content [22], suggesting that exercise-induced autophagy can potentially ameliorate pathological conditions linked to autophagy malfunction across various tissues and organs. The modulation of autophagy activity induced by exercise can be attributed to the activation of multiple signal transduction pathways, with variations in autophagy activity dependent on the mode and conditions of exercise. Therefore, using exercise as a stimulus for autophagy is a suitable and scholarly approach to prevent, manage, and rehabilitate chronic diseases by ameliorating aberrant autophagy function, ultimately promoting optimal health and preventing diseases.

## 3. Exerkine-Mediated Inter-Organ Communication Modulates Autophagy to Ameliorate Chronic Diseases

**Figure 1:**
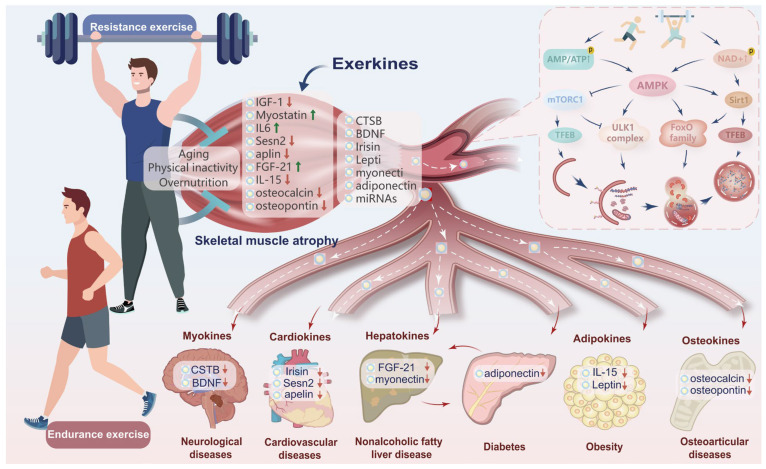
Integrated overview of exercise-induced autophagy regulation via intracellular signaling and inter-organ exerkine communication. Exercise activates autophagy through conserved molecular pathways across multiple cell types, including AMPK activation, mTORC1 suppression, and TFEB/PGC-1α-driven transcriptional networks. These signals operate in hepatocytes (AMP/ATP, NAD^+^, AMPK, SIRT1), cardiomyocytes (AMPK-SIRT1, TFEB, PGC-1α), pancreatic β cells (ULK1, FoxO, TFEB), skeletal muscle (AMPK-ULK1, FoxO3), adipocytes (AMPK-mTORC1), osteocytes (mechanosensitive AMPK/mTORC1), neurons (ULK1, Beclin1, PGC-1α), and other cells to enhance mitochondrial quality control, lipid metabolism, proteostasis, and stress resistance. Concurrently, exercise stimulates the release of exerkines from various tissues, establishing an inter-organ communication network that modulates autophagy in distant organs. Key mediators include hepatokines (FGF-21, myonectin), cardiokines (irisin, Sestrin2), myokines (myostatin, IGF-1, IL-6, IL-15, BDNF, CTSB), adipokines (leptin, adiponectin), and osteokines (osteocalcin, osteopontin). These factors fine-tune autophagic flux in target tissues to counteract pathologies such as nonalcoholic fatty liver disease, cardiovascular diseases, diabetes, obesity, osteoarticular disorders, and neurodegeneration. Thus, exercise orchestrates a multi-tissue adaptive program through cell-autonomous signals and exerkine-mediated crosstalk, restoring autophagic homeostasis and ameliorating chronic diseases.

To date, exerkines, encompassing cytokines, peptides, growth factors, and small organic acids, have been identified as potential mediators of inter-organ communication between skeletal muscle and other tissues, including the heart, brain, adipose tissue, pancreas, liver, and bone, with both positive and negative effects (Figure 1). Importantly, exerkines have been found to modulate several physiological processes, such as nutrient sensing, stress signal transduction, protein homeostasis, and metabolic regulation. Exerkines can either facilitate or impede autophagy, while conversely, autophagy can modulate the secretion of exerkines. This mutual interaction governs the progression and management of a series of diseases. Notably, the exerkine–autophagy relationship is highly context-dependent and can be markedly reshaped by nutritional state (e.g., fasted vs. fed conditions, glycogen availability, and chronic overnutrition) [23]. Fasting and low energy availability generally favor AMPK/SIRT1 activation and mTORC1 suppression, thereby facilitating autophagy initiation and substrate recycling, whereas postprandial insulin/IGF-1 signaling may transiently prioritize anabolic recovery and attenuate autophagy initiation [24]. Importantly, overnutrition and obesity can impair lysosomal function and distort autophagic flux, potentially confounding exerkine readouts and downstream organ crosstalk [25]. Future studies should therefore stratify or control for nutritional status and sampling time to improve comparability across models.

The interaction between autophagy and exerkines is bidirectional; while exerkines modulate autophagic flux, secretory autophagy mechanisms actively regulate the cargo composition of extracellular vesicles (EVs) [26]. Recent evidence indicates that autophagy proteins participate in the unconventional secretion of cytokines and post-translational modifications of EV proteins, which are critical for intercellular communication in chronic inflammatory states [27]. For example, in the context of rheumatoid arthritis, secretory autophagy has been linked to the production of citrullinated proteins that are loaded into EVs, a process that can activate the immune system and perpetuate inflammation [28]. Extending this concept to the context of physical activity, we propose that an “exercise-secretory autophagy-EV” axis may exist, representing a novel frontier in understanding how the metabolic state of one tissue influences systemic inflammation and organ crosstalk.

### 3.1. Myokines Mediate Muscle–Muscle Crosstalk Based on Autophagy

#### 3.1.1. Insulin-like Growth Factor 1 (IGF-1)

Among the various exerkines, myokines secreted by skeletal muscle itself have been most extensively studied for their autocrine and paracrine roles in regulating muscle mass and function through autophagy. IGF-1 serves as a key mediator of muscle mass regulation, exhibiting a context-dependent dual role that shifts from acute anabolic signaling to chronic age-related decline and adaptive stress responses. Acute high levels of IGF-1 inhibit autophagy via the Akt/mTORC1 pathway, while chronic low levels of IGF-1 in aging muscle lead to autophagic stagnation, and moderate IGF-1 supplementation can restore adaptive autophagic flux. During the immediate post-exercise recovery phase, acute spikes in IGF-1 activate the Akt/mTORC1 pathway to prioritize protein synthesis and muscle hypertrophy, thereby suppressing autophagy through mTOR-mediated inhibition of ULK1 phosphorylation and FoxO3-driven gene induction. This acute anabolic signaling is exemplified by decreased LC3-I to LC3-II conversion in IGF-1-treated chicken myotubes, confirming that autophagic flux is temporarily downregulated to favor tissue repair and growth [29,30].

However, in the context of aging and pathological stress, the role of IGF-1 becomes more complex and shifts toward quality control rather than simple suppression. Chronically low IGF-1 levels in aged skeletal muscle contribute to “autophagy stagnation”, impairing the clearance of damaged proteins and organelles [31]. Paradoxically, IGF-1 overexpression in mice—restoring levels that typically decline with age—has been shown to increase LC3 conversion, suggesting a restorative effect on autophagic activity [32]. Similarly, in pathological models such as cisplatin-induced atrophy, cancer cachexia, and Ang II-induced muscular dystrophy, where diminished autophagy leads to the accumulation of dysfunctional mitochondria and impaired energy metabolism, IGF-1 administration rescues muscle atrophy by activating adaptive autophagy and improving ubiquitin-proteasome system (UPS) function rather than by suppressing proteolysis [33,34,35].

Thus, IGF-1 does not simply “turn off” autophagy; rather, it fine-tunes the balance between proteolysis and protein synthesis depending on physiological context, shifting from an autophagy-suppressor in healthy hypertrophic conditions to a quality-control facilitator under metabolic stress. The conflicting findings in the literature regarding mTOR and FoxO signaling [36] underscore the challenge of capturing dynamic autophagic flux through static measurements, highlighting the critical need for dynamic flux assays to accurately characterize these complex, context-dependent processes.

#### 3.1.2. Myostatin (MSTN)

MSTN, also known as growth differentiation factor 8 (GDF-8), is a highly conserved member of the TGF-β protein family encoded by the myostatin gene. Muscle atrophy is characterized by a reduction in muscle fiber diameter and total protein content, resulting from decreased protein synthesis and excessive protein degradation. FoxO transcription factors, critical for catabolic reactions, play a pivotal role in muscle atrophy by coordinating the activation of the autophagolysosome (ASL) and the UPS. MSTN can activate FoxO1, leading to increased expression of muscle atrophy F-box (MAFbx) and muscle ring finger protein 1 (MuRF1) [37]. Interestingly, FoxO1 upregulates MSTN expression, creating a synergistic feedback mechanism that amplifies the atrophic response [38]. Additionally, high levels of MSTN expression are closely associated with the inhibition of protein synthesis and increased protein degradation, which further exacerbates the process of muscle atrophy [39].

ASL plays a crucial role in protein loss in atrophic muscles. A close correlation exists between MSTN and UPS, and up-regulation of ASL is observed in trout myotube atrophy in vitro [40]. Similarly, MSTN can induce muscle atrophy by up-regulating autophagy-related genes, MAFbx, MuRF1, and proteasome subunits in response to TNF-α [41]. MSTN can also trigger proteolysis and up-regulate Atrogin-1, MuRF1, and LC3 in the extensor digitorum longus muscle from mice and C2C12 cells [39]. Moreover, MSTN can reduce the size of myotubes by inhibiting the initiation of protein synthesis, which is crucial for muscle cell growth and development. This inhibition is mediated through the suppression of the Akt/mTOR signaling pathway, which is a key regulator of protein synthesis in muscle cells [42]. In MSTN-deficient mice, the augmented level of p70S6K, a biomarker for protein synthesis, is accompanied by an overall increase in Akt expression [43], suggesting that the MSTN and IGF-1 signaling pathways exhibit opposing effects. This implies that IGF-1 could be a potential therapeutic target for muscular dystrophy.

Resistance exercise is a potent stimulator of MSTN suppression [44], which acts as a permissive signal for autophagic remodeling. While high MSTN levels drive excessive muscle loss via FoxO1-mediated hyper-autophagy, resistance training-induced MSTN down-regulation promotes a more “efficient” autophagic flux [45]. This remodeling prevents the accumulation of dysfunctional proteins while shifting the metabolic balance toward hypertrophy, demonstrating that the axis transitions from catabolic proteolysis to adaptive quality control.

#### 3.1.3. Interleukin-6 (IL-6)

IL-6 is an early muscle hormone released into the bloodstream during muscle contraction, and its expression is directly correlated with the duration and intensity of exercise. IL-6 is predominantly expressed in type I (slow-twitch) muscle fibers [46] and elicits pro-inflammatory effects by activating cells via the trans-signaling pathway, leading to the development of chronic inflammation in skeletal muscle [47].

Currently, increasing evidence confirms that IL-6 and other pro-inflammatory agents may stimulate skeletal muscle proteolysis and insulin resistance, which are significant contributors to muscle atrophy [48]. Meanwhile, IL-6 can bind to the expressed gp130 signal transduction receptor subunit, activating the IL-6/gp130/STAT3 signaling pathway and exacerbating skeletal muscle atrophy [49]. Similarly, local inhibition of STAT3 in skeletal muscle reveals a decrease in mitophagy in denervated skeletal muscle [50]. Moreover, research has shown that IL-6 secreted by tumor cells can accelerate autophagy in myotubes, a process that involves the lysosomal degradation of cellular components, thereby contributing to muscle wasting. This autophagic activity is often mediated through IL-6 trans-signaling, where IL-6 binds to a soluble IL-6 receptor, forming a complex that can interact with gp130 on target cells, activating downstream signaling pathways such as STAT3. This pathway has been identified as a critical mediator of muscle atrophy in cancer cachexia, as it promotes the degradation of muscle proteins and inhibits muscle synthesis [51]. To date, the understanding of the underlying molecular mechanisms for the impact of IL-6 on autophagy remains limited. However, the capacity of IL-6 to activate crucial and widespread secondary messenger pathways linked to the regulation of autophagy in skeletal muscle suggests the potential of IL-6 to modulate autophagy in the context of skeletal muscle atrophy.

### 3.2. Cardiokines Mediated Skeletal-Muscle–Myocardium Crosstalk Based on Autophagy

The myocardium, an energy-sensitive organ, is regulated by normal or optimal autophagy to maintain its energy supply and function. A growing body of evidence indicates that basal autophagy is essential for preserving cardiovascular function, while excessive or inadequate autophagic flux may lead to cardiovascular diseases (CVDs) [52]. Consistent with this, recent studies have established a correlation between the quantity of human muscle tissue and the prevalence and mortality of cardiovascular diseases. Indeed, skeletal muscle atrophy resulting from aging and weightlessness is linked to anomalous cardiovascular metabolism and function [53]. Consequently, the regulation of skeletal muscle and cardiovascular function is attributed to the secretion of specific hormones, namely, myokines and cardiokines.

#### 3.2.1. Irisin

In 2012, a novel myokine known as irisin was identified as a cleaved product of its precursor protein FNDC5, produced by skeletal muscle. Spiegelman’s research group reported that PGC-1α promotes the expression of FNDC5 mRNA in skeletal muscle, leading to the formation of a 112-amino acid polypeptide fragment that is secreted into the bloodstream following cleavage and modification [54]. Notably, the myocardium is a superior source of irisin when compared with skeletal muscle [55]. Exogenous irisin treatment reduced autophagy in H9c2 cells, as evidenced by a decrease in the LC3-II/LC3-I ratio. This inhibitory effect was partially mediated by the PI3K/Akt signaling pathway [56]. Similarly, irisin enhanced the survival rate of insulin-producing cells in cultured INS-1 cells by activating the AMPK/ULK1 signaling pathway [57]. In endothelial cells, uncoupling protein 2 (UCP2) typically facilitates AMPK up-regulation [58]; however, the absence of irisin led to a significant down-regulation of UCP2 [59]. In addition to reducing ROS levels in endothelial cells, irisin can rescue autophagy disorders, inhibit apoptosis and inflammation, promote endothelial cell proliferation, and maintain endothelial homeostasis. The level of irisin can serve as a biomarker in patients with myocardial infarction (MI) to predict the probability of cell death due to acute heart failure [60]. Irisin, through the ERK signaling pathway, activates mitophagy, thereby promoting angiogenesis, reducing the area of MI, and mitigating cell damage [61]. Consequently, irisin can regulate autophagy and play a protective role in preventing and treating cardiac hypertrophy and disease-induced cardiomyopathy.

#### 3.2.2. Sestrin 2 (Sesn2)

Sestrins (Sesns) are a class of highly conserved, stress-induced proteins that respond to environmental stresses, including oxidative stress, DNA damage, and hypoxia. Sesn2 has been confirmed to activate AMPK, suppress mTORC1, and stimulate autophagic activity, promoting lifespan and health [62]. For instance, the regulation of the AMPK/mTOR axis has been demonstrated to mitigate tendon stem/progenitor cell senescence and delay tendon aging. In this study, metformin, an AMPK activator, was shown to mitigate senescence and restore functions such as proliferation and differentiation in tendon stem/progenitor cells, highlighting the therapeutic potential of targeting the AMPK/mTOR axis in age-related disorders [63]. Furthermore, Sesn2 can prevent skeletal muscle atrophy by activating autophagy, suppressing mTORC1, and downregulating ULK1 and S6 phosphorylation [64]. Therefore, Sesn2 plays a crucial role in maintaining muscular homeostasis during the aging process.

Researchers have demonstrated a significant increase in myocardial fibrosis and a decrease in capillary density in *Sesn2* knockout mice, indicating the potential adverse effects of *Sesn2* deletion on myocardial fibrosis [65]. In aging hearts, Sesn2 mitigates myocardial hypertrophy and enhances substrate metabolism through the Sesn2/mTORC1 signaling pathway [66]. Additionally, autophagy is impaired in cardiac ischemia–reperfusion injury, and the restoration of autophagosome clearance attenuates reoxygenation-induced cell death [67]. It is widely acknowledged that the myocardium becomes increasingly vulnerable to ischemia–reperfusion damage with age [68]. This susceptibility is attributed to impaired autophagy and decreased in key autophagy-related proteins and signaling pathways, such as the mTOR pathway, which is known to be dysregulated in aging hearts [69,70]. Given the potential of *Sesn2* as a therapeutic target for preventing ischemic heart disease in aging individuals, it may represent a practical and effective approach.

#### 3.2.3. Apelin (APLN)

APLN is widely expressed in skeletal muscle, heart, and adipose tissue and acts as a peptide ligand for the G protein-coupled apelin peptide jejunum, apelin receptor (APJ). The apelin receptor expressed on skeletal muscle stem cells can promote their proliferation and differentiation in vitro and in vivo, playing a crucial role in muscle regeneration. As individuals age, APLN triggers mitochondrial biogenesis via AMPK-dependent signal pathways, fosters autophagy, and mitigates inflammation, impeding the progression of sarcopenia [23]. Recent studies have demonstrated that APLN is pivotal in diverse biological functions, particularly in the cardiovascular and metabolic systems [71]. APLN has a protective role in heart failure, including acute pathologies such as MI and chronic heart disorders such as excessive fibrosis and hypertrophy [72].

Moreover, APJ-null mice exhibit a reduction in chronic pressure overload-induced myocardial hypertrophy and heart failure [73]. This observation aligns with the growing body of evidence implicating apelin in autophagy, a process crucial in the pathogenesis of chronic diseases. Specifically, apelin-13 is the predominant apelin isoform in human cardiac tissue and plays a protective role in myocardial contraction, blood pressure regulation, and myocardial injury. Alterations in heart autophagy levels have been reported in response to stress (ischemia/reperfusion) [74,75] and cardiovascular diseases (cardiac hypertrophy and heart failure). Additionally, apelin-13 has been shown to inhibit nicotine-induced apoptosis and oxidative stress in H9c2 cells via the PI3K/Akt signaling pathway, further supporting its protective role in cardiac cells [76]. This protective mechanism is also evident in apelin-13’s ability to reverse bupivacaine-induced cardiotoxicity by activating the AMPK pathway, which is known to regulate energy homeostasis and autophagy [77]. Hence, APLN could potentially serve as a mediator in the intercommunication between skeletal muscle and myocardium, representing a promising biomarker for fundamental cardiovascular disorders in the context of aging-related muscle wasting.

### 3.3. Myokines Mediate Skeletal-Muscle–Brain Crosstalk Based on Autophagy

Neurodegenerative diseases, including Alzheimer’s disease (AD) and Parkinson’s disease (PD), are characterized by the anomalous accumulation of misfolded and denatured proteins within brain tissue. These abnormal protein aggregates may originate from intracellular or extracellular compartments, neurons, or oligodendrocytes. Consequently, autophagy plays a crucial role in the clearance of these abnormal protein aggregates associated with neurodegenerative diseases [78]. On the other hand, physical activity can stimulate the production and release of myokines in metabolically active skeletal muscle, which can enhance brain function and protect against neuronal damage.

#### 3.3.1. Cathepsin B (CTSB)

CTSB, a cysteine protease associated with hippocampal function, is responsible for the degradation of proteins entering the endolysosomal system of cells through phagocytosis. One of the key aspects of lysosomal dysfunction in aging is the impairment of autophagic flux, which is the process by which cellular debris is sequestered and degraded. Studies have shown that lysosomal dysfunction can lead to an accumulation of autophagic vacuoles and a decrease in the degradation capacity of lysosomes, contributing to cellular senescence and tissue aging [79]. Abnormal lysosomes are associated with amyloid plaques formed by Aβ deposition, and the proteolytic detoxification of the Aβ42 peptide is localized to neuronal lysosomes [80]. Autophagy is activated by CTSB, the most abundant protease in lysosomes [81]. Notably, the up-regulation of CTSB is closely associated with the accumulation of Aβ, other related peptides and huntingtin protein, as well as the impairment of protein clearance pathways [82]. Transmission electron microscopy results indicate that CTSB deficiency can increase the number and size of lysosomes and autophagosomes, suggesting that CTSB may hinder the bioavailability of lysosomes and autophagosomes [83]. Similarly, cilostazol, a positive regulator of the autophagolysosomal system, enhanced CTSB activity and reduced Aβ42 accumulation in neuroblastoma cells through the AMPK/Sirt1 signaling pathway [84]. In AD transgenic mice, the administration of CTSB significantly reduced Aβ42 peptide levels and improved learning and memory capacity [85]. Furthermore, CTSB can traverse the blood–brain barrier and augment brain-derived neurotrophic factor (BDNF) and doublecortin (DCX), enhancing neuroprotection [86]. It is widely believed that autophagy and apoptosis can antagonize or assist each other, influencing cell fate, as similar pathways modulate these processes [87]. The B-cell lymphoma-2 (Bcl-2)/Beclin1 complex serves as a common regulator of intrinsic apoptosis and autophagy. More importantly, Bcl-2 family members are substrates for CTSB, an anti-apoptotic enzyme [88]. Consequently, as a neural network and brain function regulator, CTSB, acting as an exerkine, can potentially mitigate neurological diseases associated with AD by modulating autophagy activity in cells.

#### 3.3.2. Brain-Derived Neurotrophic Factor (BDNF)

As a member of the neurotrophic factor family, BDNF is a crucial mediator of neuroprotection, regulating the survival, growth, and maintenance of neurons and playing a pivotal role in synaptic plasticity, cell survival, and brain cell differentiation. Learning and memory capacity is closely associated with normal or higher hippocampal function, involving high-level expression of BDNF. Memory deficit, a typical characteristic of AD, is due to low BDNF levels in the blood and brain [89]. In specific contexts, a reduction in autophagy is essential for enhancing cognitive performance [90]. Increasing studies have demonstrated that BDNF regulates synaptic plasticity through the activation of mTOR via the interaction between its receptor kinase B (TrkB) and PI3K, resulting in the inhibition of autophagy [91]. On the one hand, BDNF stimulates long-term potentiation (LTP), a critical mechanism for memory and learning. On the other hand, the presence of the BDNF receptor on the autophagy membrane suggests that autophagy may also play a role in BDNF regulation. BDNF is a crucial mediator of activity-dependent synaptic strength modification, promoting the augmentation of synaptic vesicles and neurotransmitter release. Moreover, autophagy significantly contributes to BDNF-mediated synaptic plasticity. When BDNF is absent, autophagy becomes hyperactive, leading to synaptic malfunction and impaired LTP [90]. Stress signaling triggers autophagy, releasing cathepsins and resulting in the accumulation of BDNF in the mouse brain [92]. Furthermore, BDNF may exert a neuroprotective function through the up-regulation of p62 in primary cortical neurons [93], inhibiting 3-NP-induced autophagy, and may also be implicated in BDNF expression in microglia [94]. Autophagy-mediated regulation of neuroplasticity is facilitated by BDNF signaling, which may be altered by autophagy activation in response to intracellular reorganization requirements. Taken together, the available experimental evidence suggests that BDNF can modulate neuroplasticity by modulating autophagy, influencing the pathogenesis of neurodegenerative disorders.

### 3.4. Adipokines Mediate Skeletal-Muscle–Adipose Crosstalk Based on Autophagy

The deposition of adipose tissue is primarily caused by overnutrition, lack of exercise, and heredity, leading to systemic lipotoxicity through the cytotoxic effect of excessive lipids, which inhibits the formation of autophagosomes and affects autophagy activity [95]. Skeletal muscle and adipocytes contain metabolically active proteins, referred to as exerkines and adipokines, while adipo-myokines, released by skeletal muscle and adipose tissue, exert paracrine and autocrine effects on organs. Autophagy has been demonstrated to facilitate certain biological processes of adipokines, establishing adipokines, such as interleukin-15 (IL-15), Adiponectin (ADPN), and leptin, as intermediaries between adipose tissue and skeletal muscle.

#### 3.4.1. IL-15

During exercise training, IL-15 can accumulate in skeletal muscle and exert its effects on adipose tissue via the bloodstream, regulating adipose tissue metabolism and contributing to the formation of the skeletal-muscle–fat axis [96]. In the context of sarcopenia, IL-15 promotes skeletal muscle growth and reduces adipose mass, making it a potential treatment for skeletal muscle loss. Reduced levels of IL-15 protein in skeletal muscle and serum are associated with sarcopenia in the elderly, while controls exhibit a significant increase in IL-15 levels when compared to sarcopenic individuals [97]. Notably, independent centenarians exhibit significantly elevated serum IL-15 concentrations, implying a protective effect against aging-related debility [98]. Current evidence suggests that decreased IL-15 levels are linked to sarcopenia, obesity, and other pathologies. IL-15 can mitigate adipogenesis in adipose tissue, enhance fatty acid mobilization, and stimulate the differentiation of muscle satellite cells, serving as a significant regulatory factor in the coculture of skeletal muscle cells and adipocytes [99]. Similarly, mice overexpressing IL-15 experience a significant reduction in body weight when compared to wild-type counterparts, while IL-15 knockout mice exhibit a marked increase in body fat. However, exogenous IL-15 supplementation can mitigate this phenomenon [100]. Consequently, IL-15 has the potential to enhance the rate of lipid catabolism within the body, reducing lipid accumulation in tissues. Inhibition of IL-15 results in the inhibition of FoxO1 phosphorylation, leading to FoxO1 translocation to the cytoplasm and subsequent inhibition of autophagy. It has been reported that cytoplasmic FoxO1 undergoes phosphorylation and interacts with *Atg*7 to induce autophagy. Cells harboring FoxO1 mutations exhibit inhibited autophagy, resulting in compromised cell growth and viral clearance [101]. These findings suggest that IL-15 serves as a crucial mediator in the skeletal-muscle–adipose interplay by regulating autophagy, although the precise molecular mechanism remains undetermined.

#### 3.4.2. Adiponectin

Adiponectin, the most prevalent adipokine secreted by adipocytes, possesses diverse biological functions. Its receptors, AdipoR1 and AdipoR2, are primarily distributed in skeletal muscle and liver, respectively. AdipoR1 activation in skeletal muscle stimulates AMPK, reduces gluconeogenesis, and promotes fatty acid oxidation. AdipoR2 activation in the liver triggers PPAR-γ, which inhibits lipid deposition, oxidative stress, and inflammation [102]. For example, a correlation exists between the myopathic phenotype and dysfunctional autophagy, while globular adiponectin induces autophagy in myoblasts, indicating that adiponectin plays a role in inducing autophagy in skeletal muscle [103]. Adiponectin has also been found to restore insulin sensitivity in insulin-resistant skeletal muscle. Remarkably, this effect is facilitated by the restoration of autophagy, which is absent in *Atg*5-dominant negative cells with deficient autophagy [104].

The protective effect of adiponectin on liver cells against the cytotoxic effects of ethanol is attributed to its capacity to induce autophagy [105]. Ethanol reduced autophagy-related gene expression and autophagosome formation in hepatocytes, directly related to toxicity. Interestingly, autophagy activation has been found to ameliorate several other liver diseases, such as alcoholic and nonalcoholic fatty liver disease (NAFLD) [106], which can be rescued through the therapeutic administration of adiponectin [107]. Therefore, it is plausible that the protective role of adiponectin in various forms of acute and chronic liver injury is mediated by induced autophagy, representing a common mechanism. Additionally, adiponectin induces autophagy activation in rat hepatocytes treated with acetaminophen, playing a critical role in suppressing endoplasmic reticulum stress and inflammasome activation, safeguarding hepatocytes against acetaminophen-induced cell death [108]. These findings indicate that adiponectin-mediated autophagy plays a crucial role in protecting against liver disease.

#### 3.4.3. Leptin

The hormone leptin, primarily synthesized by white adipose tissue, is secreted in proportion to the amount of body fat. Leptin-deficient ob/ob mice develop obesity, hyperinsulinemia, and hyperglycemia, mirroring the metabolic syndrome (MetS) observed in humans with leptin deficiency [109]. Previous studies have established a correlation between circulating leptin levels and *Atg*5 mRNA expression in visceral adipose tissue (VAT) [110]. Recent studies have demonstrated that leptin can induce autophagy in various tissues, including the myocardium, kidney, skeletal muscle, and liver, as evidenced by increased expression of LC3 and decreased expression of p62 proteins following leptin injections [111]. Hypothalamic knockout of the *Atg*7 gene has demonstrated a correlation among leptin resistance, weight gain, and obesity [112]. Furthermore, *Atg*7 knockout mice showed leptin resistance due to the failure of STAT3 activation by leptin [113]. Notably, leptin may be involved in the dysregulation of adipocyte autophagy in obesity [114]. In VAT, obesity is associated with an increase in autophagic flux. The impairment of lipid metabolism, dysfunction of adipose tissue, and obesity are not directly interrelated; however, autophagy plays a significant role in adipocyte maturation through lipolysis. Taken together, these results imply that obesity exerts autocrine/paracrine effects through the activation of autophagy in adipose tissue via leptin signaling.

### 3.5. Hepatokines Mediate Skeletal-Muscle–Hepatocyte Crosstalk Based on Autophagy

The liver is a vital organ for energy storage and plays a crucial role in the metabolism of glucose and lipids throughout the body. Hepatic fat content serves as a reliable indicator of metabolic abnormalities, with hepatic steatosis being causally linked to NAFLD, MetS, and type 2 diabetes mellitus (T2DM) [115]. At the same time, the abnormal functional status of autophagy is associated with obesity and atherosclerosis and is indispensable for survival [116]. The global deletion of *Atg*5 and *Atg*7 in mice results in perinatal lethality [117], likely due to an inability to catabolize liver proteins and glycogen. Hepatokines, which are novel hormones synthesized by the liver tissue, exhibit the potential to either exacerbate or ameliorate metabolic conditions and are subject to modulation via autocrine, paracrine, and endocrine mechanisms in both hepatic and extrahepatic tissues.

#### 3.5.1. Fibroblast Growth Factor 21 (FGF-21)

FGF-21, a hormone that mimics the effects of starvation, plays a multifaceted role in metabolic processes, particularly in aiding tissues to cope with nutrient deficiency. It is produced by multiple organs—most notably the liver, but also skeletal muscle and adipose tissue—in response to cellular stress [118]. In the context of exercise, FGF-21 is released from both hepatic and muscular sources and has been identified as a potential biomarker for mitochondrial disorders in skeletal muscle. A positive correlation between serum FGF-21 levels and sarcopenia further supports this notion [119]. In fasted FGF-21-knockout mice, muscle protection is not dependent on the UPS but relies on autophagy and mitophagy pathways [120]. Bnip3, a critical factor in FGF-21-induced muscle loss, is implicated in the removal of damaged mitochondria through mitophagy [120]. It is noteworthy that FGF-21 is currently recognized as a hepatic factor that plays a crucial protective role in glycolipid metabolism and the onset and progression of NAFLD [121]. FGF-21 directly modulates lipid metabolism and inhibits hepatic lipid accumulation independently of insulin [122]. In addition to its impact on lipid metabolism, FGF-21 enhances insulin sensitivity and reduces fasting glucose levels in NAFLD mouse models [123]. Conversely, adenovirus-transfected FGF-21 knockout mice are more susceptible to hepatic lipidosis and hyperlipidemia [124]. Additionally, autophagy deficiency in skeletal muscle protects against obesity and insulin resistance by upregulating FGF-21 production [125]. Mechanistically, impaired autophagy is linked to mitochondrial dysfunction, leading to the release of FGF-21. Recent findings suggest that FGF-21 may alleviate CCl4-induced acute liver injury by inducing autophagy, a process mediated by SIRT1 [126]. Furthermore, *Atg*7 knockout can confer protection against HFD-induced obesity and glucose intolerance [127]. These studies collectively demonstrate that FGF-21 regulates autophagy activity, potentially offering therapeutic benefits for skeletal muscle atrophy, obesity, and NAFLD.

#### 3.5.2. Myonectin

Myonectin, a member of the C1q/TNF-related protein family, has been identified as a regulator of glucose and fatty acid metabolism. It is a novel, nutrition-responsive myokine that was highly induced in differentiated myotubes and predominantly expressed by skeletal muscle [128]. Myonectin can ameliorate skeletal muscle dysfunction through AMPK/PGC1α-dependent mechanisms, suggesting that myonectin could represent a therapeutic target of muscle atrophy [129]. In adipose tissue and the liver, myonectin stimulates fatty acid uptake, reducing plasma-free fatty acid levels. This effect is believed to be mediated by an increase in scavenger and transporter proteins, including CD36, FATP-1, and FABP-4 [130]. However, myonectin does not appear to influence adipocyte lipolysis or glucose homeostasis [131]. Based on these observations, it has been suggested that myonectin may serve as an indicator of nutrient status and facilitate nutrient assimilation and retention. Furthermore, myonectin inhibits autophagy by activating the PI3K/Akt/mTOR signaling pathway in the liver [132]. Myonectin can enhance hepatocyte lipid uptake by upregulating Cav1 and Fabp1 genes, which are involved in fatty acid absorption [131]. Plasma myonectin levels correlate positively with insulin resistance, and myonectin secretion may serve as a compensatory mechanism for insulin resistance in humans [133]. Additionally, previous studies in genetic mouse models have shown that myonectin deficiency induces lipid intolerance associated with HFD feeding, altering fat distribution in the tissues and liver [134]. These findings suggest that myonectin acts as a hepatokine, linking skeletal muscle with lipid metabolism in the liver and adipose tissue.

### 3.6. Osteokines Mediate Skeletal-Muscle–Bone Crosstalk Based on Autophagy

Muscles and bones interact to maintain their structural and functional integrity. It is now recognized that muscles and bones engage in bidirectional communication, exchanging biochemical signals that influence the metabolism of both tissues and the entire organism. This communication occurs via the endocrine system, regulated by cytokines such as myokines and osteokines. The skeletal system undergoes a lifelong process of bone formation and resorption, which is essential for maintaining homeostasis. The balance between osteoblasts and osteoclasts is dynamic and is critical in this process. The regulation of osteoblast–osteoclast coupling involves numerous cellular functions and molecular signaling pathways, including the newly discovered mechanism of autophagy-mediated bone remodeling and regeneration [135]. Autophagy is implicated in preosteoblast differentiation, osteoblast–osteocyte transition, and the genesis and function of osteoclasts [136]. Furthermore, the inhibition of autophagy in osteocytes leads to bone tissue senescence [137]. These findings collectively demonstrate the critical role of autophagy in bone remodeling and regeneration.

#### 3.6.1. Osteocalcin (OC)

OC has multiple functions, including glucose metabolism, energy metabolism, and ectopic calcification, and can exert a systemic effect on the body by affecting both skeletal muscle and bone [138]. Studies published in 2016 indicate that OC levels increase in mice and humans during physical activity and aging [139]. Notably, acute or chronic administration of exogenous OC can enhance exercise capacity in mice at different age stages. OC can upregulate fatty acid transporters, enhance β-oxidation, facilitate glucose uptake, and stimulate catabolism in skeletal muscle [140]. Additionally, OC and autophagy have been linked to various physiological and pathological processes. For instance, autophagy has been shown to regulate OC expression through time-dependent modulation of mTOR signaling during osteogenic differentiation. Autophagy inducers suppress OC expression in osteoblasts derived from mouse calvaria while inhibiting cAMP expression can reverse this effect. A previous study suggested that OC can enhance insulin sensitivity by suppressing autophagy through the Akt/mTOR signaling pathway [141]. The available evidence suggests that OC may exert a protective effect on lipid and glucose homeostasis by enhancing mitochondrial activity through the correction of impaired autophagy in both adipose tissue and skeletal muscle.

#### 3.6.2. Osteopontin (OPN)

OPN, a pleiotropic glycoprotein, is widely expressed in various bodily tissues, including bone, immune cells, smooth muscle, neurons, adipocytes, and Kupffer cells. OPN is secreted by skeletal muscle myoblasts and macrophages, and its expression is strongly upregulated in response to muscle damage [142]. Consistent with this, OPN secretion in mice with muscular dystrophy may contribute to inflammation during muscle regeneration [143]. Additionally, OPN levels are higher in the bones of younger individuals compared to older individuals [144]. Further studies have confirmed a statistically significant elevation in serum OPN levels among menopausal women compared to women of reproductive age [145]. In mice with osteoarthritis, the absence of OPN leads to a decrease in proteoglycan levels, an increase in MMP-13 release, and a reduction in chondrocyte count and subchondral osteoarthritis [146]. Previous investigations have established the significance of chondrocyte proliferation in OA [147]. The administration of rapamycin can reduce cartilage degradation in an OA model, associated with a decrease in the expression of disintegrin-like and A disintegrin and metalloproteinase with thrombospondin type 1 motif 5 (ADAMTS-5) and inflammation in synovial tissue, achieved by activating autophagy in chondrocytes [148]. Furthermore, the MAPK signaling pathway is involved in the promotion of chondrocyte proliferation and the suppression of autophagy activity by OPN [149]. As a mediator of physiological and pathological processes, OPN regulates the aging of various tissues and organs.

### 3.7. Integration of the Exerkine–Autophagy Axis with Hallmarks of Aging

The exercise–exerkine–autophagy axis serves as a systemic coordinator that directly mitigates several recognized hallmarks of aging by fine-tuning autophagic flux across multiple tissues [150]. Specifically, this axis counteracts loss of proteostasis through exerkines such as MSTN and IGF-1, which regulate autophagic clearance of misfolded proteins in skeletal muscle, while BDNF and CTSB promote degradation of protein aggregates in neuronal cells to preserve neuroprotection [151,152]. Regarding mitochondrial dysfunction, factors including Irisin and Sesn2 enhance mitophagy via AMPK-dependent pathways to ensure a healthy mitochondrial pool and reduce oxidative stress, with APLN additionally triggering mitochondrial biogenesis and autophagy to impede sarcopenia [23,153]. The axis also modulates deregulated nutrient-sensing by influencing key pathways such as AMPK/mTOR and SIRT1; for instance, FGF-21 and adiponectin restore metabolic flexibility and energy homeostasis by correcting impaired autophagy in adipose and liver tissues [154]. Finally, by functioning as systemic messengers—including circulating miRNAs—exerkines synchronize autophagic status across distant organs, thereby slowing the progression of systemic senescence and age-related chronic diseases through altered intercellular communication [155]. Thus, the exerkine–autophagy axis provides a mechanistic link through which exercise simultaneously counteracts multiple facets of age-related cellular decline.

## 4. MiRNAs as Critical Players of Exerkines in Crosstalk Among Multiple Tissues

**Figure 2:**
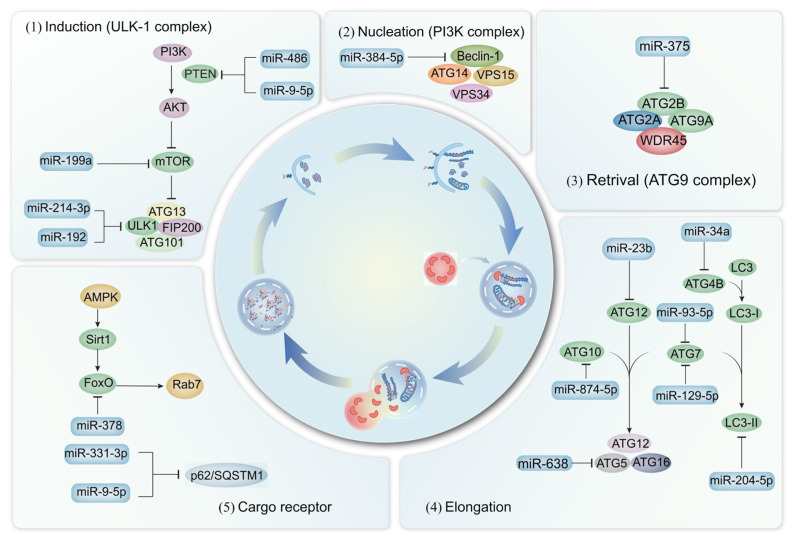
Schematic depiction illustrating the roles of relevant miRNAs during the core phase of autophagy. Key miRNAs modulate distinct autophagy stages, induction (miR-199a, miR-214-3p), nucleation (miR-486, miR-9-5p, miR-375), retrieval (miR-128a, miR-93-5p, miR-204-5p), and elongation, targeting core autophagy components (ULK1, PI3K, ATG7, p62). These miRNAs fine-tune autophagy networks—via PI3K/Akt, mTOR, and TLR4 pathways—to mitigate chronic diseases: miR-638 and miR-384-5p suppress cardiac autophagy to protect against infarction; miR-331-3p and miR-9-5p enhance Aβ clearance in neurodegeneration; miR-204-5p and miR-129-5p regulate lipid metabolism in diabetes and NAFLD; miR-27a and miR-375 modulate chondrocyte autophagy in osteoarthritis. By coordinating inter-organ crosstalk, miRNAs serve as exerkines, balancing autophagic flux to combat metabolic, cardiovascular, and degenerative pathologies.

In addition to the well-established categories of myokines, cardiokines, adipokines, hepatokines, and osteokines, miRNAs represent a relatively new class of regulators that have emerged in the past three decades. As a class of small noncoding RNAs, miRNAs dramatically increase the complexity of inter-organ crosstalk in metabolic control by acting as stable, circulating messengers that can simultaneously regulate the expression of multiple target genes in distant tissues [156]. Serving as key intercellular communicators, miRNAs are secreted from various cell types throughout the body and either transferred to body fluids or packaged in nanovesicles. The expression of miRNAs is modulated by various stimuli, including metabolites and myokines, thereby regulating inter-organ communication and metabolic control through the release of signaling factors. The underlying mechanisms for a series of miRNAs involved in autophagy in the context of chronic diseases are also elucidated (Figure 2).

Multiple experiments have demonstrated the involvement of miRNAs in the regulation of autophagy. Specifically, miR-486 targets phosphatase and tensin homolog deleted on chromosome ten (PTEN), leading to the activation of the PI3K/Akt signaling pathway and the indirect inhibition of the Hippo signaling pathway in sarcopenia [157]. Additionally, the absence of miR-378 in mice results in compromised autophagy, abnormal mitochondrial accumulation, and excessive apoptosis in skeletal muscle, negatively impacting muscle mass and exercise capacity. Mechanistically, miR-378 facilitates autophagy initiation through the mTOR/ULK1 signaling pathway and sustains autophagy via FoxO-mediated transcriptional reinforcement by targeting phosphoinositide-dependent protein kinase 1 (PDK1) [158].

Numerous miRNAs have been identified as key regulators of autophagy during acute MI [159,160,161]. Specifically, miR-638 and miR-384-5p modulate the expression of *Atg*5 and activate the PI3K/Akt signaling pathway, respectively, leading to reduced autophagy and myocardial protection [159,160]. Furthermore, both in vitro and in vivo studies demonstrate that miR-93-5p prevents myocardial injury by targeting *Atg*7-mediated autophagy and TLR4-mediated inflammation [161]. The established association between heart hypertrophy and dysregulated cardiac autophagy extends to the cellular level. Within cardiomyocytes, miR-199a inhibits autophagy by targeting the glycogen synthase kinase 3β (GSK-3β)/mTOR complex [162]. Notably, the role of miRNAs in regulating autophagy extends beyond the heart. For example, inhibition of miR-331-3p and miR-9-5p in neurons leads to accelerated Aβ clearance and improved cognitive performance, likely by targeting SQSTM1/p62 and OPTN [163]. Additionally, miR-23b overexpression in neurons reduces apoptosis, injury, and cognitive dysfunction [164]. Consistent with these findings, miR-874-5p attenuates MPP+-induced neuronal damage in PD by targeting *Atg*10 [165].

Diabetic retinopathy (DR) is a chronic, progressive complication of diabetes. A study revealed that miR-204-5p could serve as a novel and effective treatment target for DR by inhibiting autophagy through the down-regulation of LC3-II [166]. In db/db mice, miR-129-5p impedes adipocyte differentiation and white adipocyte browning by targeting the autophagy signaling network associated with *Atg*7 [167]. Compared to obese animals and patients with NAFLD, miR-34a levels are elevated, while *Atg*4b and Rab-8b levels are concurrently decreased [168]. Furthermore, inhibiting miR-214-3p can target Ulk1, augmenting autophagy and mitigating NAFLD progression [169]. Similarly, in mice fed an HFD, miR-30a-mediated suppression of *Atg*6 can impede the protective benefits of endothelial cell autophagy [165].

Multiple miRNAs have been identified as potential contributors to the pathogenesis of OA. For instance, miR-375 expression is elevated, while *Atg*2b expression is decreased. The suppression of *Atg*2b by miR-375 impairs autophagy and increases endoplasmic reticulum stress, thereby exacerbating OA progression [170].

## 5. Physical Exercise-Derived Exerkines in Chronic Diseases Based on Autophagy

The objective of this article is to elucidate the correlation between exercise variables and chronic diseases, thereby underscoring the crucial role of exercise. Further research is imperative to clarify the specific forms of exercise, along with their optimal duration, frequency, and intensity, required to achieve desired outcomes. Additionally, diverse intensities, types, and durations of exercise induce varying levels of myokine release. Exerkines, released during skeletal muscle contraction in response to exercise, have emerged as potential therapeutic targets for chronic diseases due to their beneficial effects. Exercise-derived exerkines have been shown to influence autophagy biomarkers and other signaling pathways implicated in chronic diseases (Table 1).

MSTN exerts a negative regulatory effect on skeletal muscle size. Concurrently, endurance exercise significantly enhances the force-generating capacity of skeletal muscle in myostatin-deficient mice, accompanied by an increase in the autophagy-related gene Bnip3 [171]. A 9-week resistance training regimen promotes the progression of sarcopenia by activating the IGF-1 and its receptors, Akt/mTOR, and Akt/FoxO3 signaling pathways, thereby augmenting autophagy and reducing apoptosis in skeletal muscle of rats [172]. Additionally, high-intensity interval training (HIIT) activates the AMPK signaling pathway, upregulating proteins associated with mitochondrial biogenesis and mitophagy. This process facilitates the assembly and formation of mitochondrial super-complexes mediated by IL-15, enhancing mitochondrial function in aging skeletal muscle [173]. In senescence-accelerated SAMP mice, exercise has been shown to enhance skeletal muscle strength and autophagic activity through the activation of the adiponectin/AdipoR1-axis-mediated AMPK/FoxO-dependent mechanisms [174]. Similarly, aerobic exercise has been found to alleviate abnormal autophagy in the brains of APP/PS1 mice by upregulating AdipoR1 levels, which in turn reduces amyloid-beta deposition and associated AD-like abnormalities [175]. Prolonged HIIT can enhance the communication pathways linking adiponectin and AMPK, inducing autophagy, reducing oxidative stress, improving mitochondrial function, suppressing apoptosis, and ultimately mitigating aging-related skeletal muscle atrophy [176].

Short-term exercise activates BDNF signaling and autophagy in the hippocampal region, promoting spatial learning and memory retention [177]. Additionally, lifelong aerobic exercise enhances autophagy activity and BDNF/Akt signaling specificity in skeletal muscle, reducing inflammation and aging-related decline in exercise performance [178]. Exercise can restore motor function in mice with PD by increasing autophagy and CTSB and improving dopaminergic markers while decreasing α-synuclein levels [179]. Long-term exercise significantly reduces ROS production, potentially preventing CTSB release and lysosomal membrane permeabilization. This leads to the fusion of lysosomes and autophagosomes, forming autophagosomes that complete the cytoprotective autophagic process, ultimately alleviating the pathology of NAFLD and suppressing apoptosis and inflammation [180].

In the white adipose tissue of obese individuals consuming an HFD, exercise training enhances autophagosome formation by upregulating cathepsin L (CTSL) and downregulating CTSB [181]. Similarly, prolonged physical exercise in HFD-induced mice can induce autophagy and upregulate Sestrin2 protein in skeletal muscle [182]. In elderly mice, physical exercise enhances insulin sensitivity by stimulating the accumulation of Sestrin2 and the autophagic response in skeletal muscle [183]. Another study suggested that exercise could positively impact fatty liver and insulin resistance associated with obesity by stimulating FGF-21 and autophagy [184]. However, these findings from animal models are not fully consistent with in vivo data obtained from human studies and other rodent disease models. This discrepancy underscores the need for further research, including the use of tissue-specific knockout mice, to elucidate the role of autophagy in diverse disease states.

This article focused on the physiology of exercise-induced myokines and autophagy, exploring their proteins and their interplay during exercise interventions. This inquiry contributes to the development of molecular exercise physiology and the understanding of the pathological progression of chronic diseases. To summarize, exercise induces numerous myokines that execute various physiological functions in multiple tissues or facilitate communication among multiple tissues and organs. In-depth exploration is necessary to determine whether there is stratification or redundancy in the functions of exercise-induced myokines. Such an understanding may be beneficial for identifying crucial myokines that can be targeted for effective therapy and mimic interventions with the advantageous effects of exercise on metabolic homeostasis.

**Table 1:** Exercise-derived exerkines in chronic diseases based on autophagy and corresponding signaling pathways.

Exerkines	Model	Exercise Mode	Exercise Duration and Frequency	Autophagy-Related Markers	Relevant Biomarker/Signal Pathway	Reference
MSTN	MSTN^−/−^ mode; C57BL/6 mice; Skeletal muscle	Voluntary wheel running; Swimming	60 min; 5 times/week; 5 weeks	Bnip3 ↑	Ucp3, Cpt1α, Pdk4, Errγ ↑	[171]
IGF-1	SD rat; 18–20 months old; Skeletal muscle	Resistance training	10 repetitions/day; 3 times/week; 9 weeks	Beclin-1, *Atg*5/12, *Atg*7 ↑; LC3-II/LC3-I, p62 ↓	Akt/mTOR; Akt/FoxO3a	[172]
Sesn2	HFD-fed C57BL/6 mice; 4 weeks old; Skeletal muscle	Treadmill running	60 min; 5 times/week; 6 weeks	LC3-II/LC3-I, p-ULK1 ↑	AMPK/Sesn2	[182]
	C57BL/6 mice; 24 months; Skeletal muscle	Swimming exercise	One-time	p-ULK1, *Atg*5, *Atg*7, LC3-II/LC3-I ↑	-	[183]
BDNF	C57BL/6 mice; 12 months; Hippocampus	Running wheels	14 days	LC3-II ↑	DBHB ↑	[177]
	SD rat; 26 months old; Skeletal muscle	Treadmill running	45 min; 7 times/week; 18 months	LC3-II/LC3-I ↑	BDNF/Akt	[178]
CTSB	C57BL/6 mice; 7 weeks old; PD	Endurance exercise	60 min; 5 times/week; 6 weeks	LC3-II, p62, beclin-1 ↑	CuZnSOD, Cat, DJ1, GPX1/2, HO-1, PRXIII ↑	[179]
	SD rat; 8–10 weeks old; NAFLD	Running on rotarod	30 min; 7 times/week; 4 weeks	Beclin-1, *Atg*5, LC3II ↑	p-Akt/mTOR	[180]
	C57BL/6 mice; 5 weeks old; Obesity	Treadmill running	40 min; 5 times/week; 7 weeks	LAMP2, *Atg*7 ↑	ROS ↓	[181]
IL-15	SD rats; 18 months old; Skeletal muscle	HIIT	45 min; 5 times/week; 8 months	LC3-II/LC3-I, Beclin-1 ↑	AMPK ↑	[173]
	SAMP10 mice	Treadmill running	45 min; 3 times/week; 16 months	p-Akt, p-mTOR, Bcl-xL, p-FoxO3 ↑	p-AMPKα, p-ERK1/2, PGC-1α,adpoR1,COX-IV ↑	[174]
ADPN	24 weeks; APP/PS1 Mice	Treadmill running	45 min; 5 times/week; 12 weeks	mTOR ↓, Beclin1, P62 mRNA ↑ LC3-II/I, p62 ↑	p-AMPK/AMPK ↑ ADPN/AdipoR1 ↑	[175]
	SD rat; 18 months old; Skeletal muscle	HIIT	7 times/week; 8 months	*Atg*3, Beclin-1, LC3-II/LC3-I ↑	ADPN/AMPK	[176]
FGF-21	SD rat; NAFLD; Serum/liver	Swimming exercise	30 min; 5 days/week; 8 weeks	LC3-II/LC3-I ↑; p62 ↓	-	[184]

Note: Upward arrows (↑) denote upregulation, and downward arrows (↓) denote downregulation.

## 6. Conclusions and Future Perspectives

Over the past two decades, numerous studies have delved into inter-organ communication and its influence on metabolic homeostasis in both health and disease. Skeletal muscle, with its significant secretory function, has emerged as a key player in this intricate multidirectional crosstalk. Traditionally, exerkines released by tissues are considered the primary mediators of inter-organ communication. However, recent studies suggest a more complex hierarchical structure. Intracellular organelles, such as autophagosomes, may indirectly regulate organ communication, although the underlying mechanisms remain elusive. Furthermore, autophagy appears to impact crosstalk in overused tissue. Circulating miRNAs add complexity to this cellular communication network. A key challenge is integrating these diverse levels of inter-organ communication to identify novel targets for metabolic homeostasis.

Due to the complex and persistent nature of the pathogenesis and the involvement of muscle-secreted factors, a single biomarker cannot effectively diagnose and treat this condition. A multi-target approach would combine multiple potent skeletal-muscle-secreted factors as biomarkers and drug targets. This study focuses on screening and assessing representative and efficacious biomarkers and utilizing these biological factors to prevent and treat chronic diseases through exercise interventions. Although numerous skeletal-muscle-secreted factors implicated in chronic disease have been identified in recent studies, the underlying mechanisms remain incompletely understood. Therefore, there is a critical need to explore the functions of skeletal-muscle-secreted factors in chronic diseases and elucidate their clinical efficacy and corresponding molecular mechanisms. In summary, impaired autophagy in specific organs significantly impacts the onset and progression of various chronic diseases. Exerkines may serve as potential diagnostic biomarkers and therapeutic targets to improve the management of chronic diseases. However, a comprehensive understanding of exerkine-mediated autophagy remains elusive, and further research is therefore warranted to develop dynamic autophagy assessment frameworks centered on autophagic flux, which integrate the measurement of lysosomal function with multi-omics analyses to minimize biases arising from the sole reliance on static biomarkers, thereby clarifying the roles of autophagy-related crosstalk mediators such as exerkines in the prevention and management of chronic diseases.

## Data Availability

No new data were created or analyzed in this study.

## Abbreviations

The following abbreviations are used in this manuscript:

AD	Alzheimer’s disease
ADAMTS-5	A disintegrin and metalloproteinase with thrombospondin type 1 motif 5
ADPN	Adiponectin
Akt	Protein kinase B
AMPK	AMP-activated protein kinase
APJ	Apelin receptor
APLN	Apelin
ASL	Autophagolysosome
Bcl-2	B-cell lymphoma-2
BDNF	Brain-derived neurotrophic factor
CuZnSOD	Copper/zinc superoxide dismutase
CTSB	Cathepsin B
CTSL	Cathepsin L
CVDs	Cardiovascular diseases
DCX	Doublecortin
DR	Diabetic retinopathy
FGF-21	Fibroblast growth factor 21
FoxO	Forkhead box O
GPX	Glutathione peroxidase
GDF-8	Growth differentiation factor 8
GSK-3β	Glycogen synthase kinase 3β
HFD	High-fat diet
HO-1	Heme oxygenase-1
HIIT	High-intensity interval training
IGF-1	Insulin-like growth factor 1
IL-6	Interleukin-6
IL-15	Interleukin-15
LTP	Long-term potentiation
MAFbx	Muscle atrophy F-box
MetS	Metabolic syndrome
MI	Myocardial infarction
miRNAs	microRNAs
MSTN	Myostatin
mTORC1	Mammalian target of rapamycin complex 1
MuRF1	Muscle ring finger protein 1
NAFLD	Nonalcoholic fatty liver disease
OC	Osteocalcin
OPN	Osteopontin
P62/ SQSTM1	Sequestosome-1
PD	Parkinson’s disease
PDK1	Phosphoinositide-dependent protein kinase 1
PGC-1α	Peroxisome proliferator-activated receptor gamma coactivator 1 alpha
PRXIII	Peroxiredoxin III
ROS	Reactive oxygen species
SIRT1	Sirtuin 1
Sesn2	Sestrin 2
T2DM	Type 2 diabetes mellitus
TFEB	Transcription factor EB
TrkB	Receptor kinase B
UCP2	Uncoupling protein 2
ULK1	Unc-51-like autophagy activating kinase 1
p-ULK1	Phosphorylated ULK1
UPS	Ubiquitin-proteasome system
VAT	Visceral adipose tissue
